# Global $O(t^{-\alpha })$ stabilization of fractional-order memristive neural networks with time delays

**DOI:** 10.1186/s40064-016-2374-3

**Published:** 2016-07-09

**Authors:** Ling Liu, Ailong Wu, Xingguo Song

**Affiliations:** College of Mathematics and Statistics, Hubei Normal University, Huangshi, 435002 China; School of Mechanical Engineering, Southwest Jiaotong University, Chengdu, 610031 China

**Keywords:** Fractional-order systems, Memristive neural networks, Global $O(t^{-\alpha })$ stabilization, 37B25, 34A08

## Abstract

This article is concerned with the global $O(t^{-\alpha })$ stabilization for a class of fractional-order memristive neural networks with time delays (FMDNNs). Two kinds of control scheme (i.e., state feedback control law and output feedback control law) are employed to stabilize a class of FMDNNs. Several stabilization conditions in form of algebraic criteria are presented based on a new fractional-order Lyapunov function method and Leibniz rule. Some examples are given to substantiate the effectiveness of the presented theoretical results.

## Background

Recently, the fractional calculus serving the fractional-order models develops fast in both theoretical and application. The analysis about fractional-order models has attracted increasing attention cause of its promising applications in various areas of science and engineering (see Chen and Chen 2015; Chen et al. 2014; Liu et al. 2015; Liang et al. 2015; Li et al. 2015; Rakkiyappan et al. 2014, 2015b; Stamova 2014; Velmurugan and Rakkiyappan 2016; Velmurugan et al. 2016; Wang et al. 2014; Wu and Zeng 2016; Wu et al. 2016). Comparing with integer-order systems, fractional-order systems show the superiority of describing and modeling the real world or the practical problems such as anomalous diffusion, signal processing, fractal theory and continuum mechanics. Whereas, it is arduously to promote the development of research about fractional-order models for the absence of efficient mathematical tools. As mentioned by Chen and Chen (2016), Chen et al. (2014), some new and useful methods for the qualitative analysis of fractional-order models are very imperative.

On the other hand, memristor is a circuit element which was proposed by Chua (1971) and has been realized the prototype by Hewlett-Packard laboratory in Strukov et al. (2008) and Tour and He (2008). Different from classical resistors, memristor is a nonlinear resistor which owns non-uniqueness values. In addition, the memristor can manage and store a great quantity of information. For its excellent properties about memory, we can build a new model if the conventional resistors are replaced by the memristors in neural networks, which is called memristive neural networks. Some representative works studied on the properties of the memristive systems display its applicability in several interdisciplinary areas (see Bao and Zeng 2013; Guo et al. 2015; Wang et al. 2003; Wu et al. 2012; Wu and Zeng 2012; Wen and Zeng 2012; Zhao et al. 2015). From the description of memristive neural networks, combining memristors with infinite memory is extremely interesting. An advantage of fractional-order systems in comparison to integer-order systems is that fractional-order systems can generate infinite memory. Therefore, merging the memristors into a class of fractional-order neural networks is pretty anticipated. Although stability analysis of fractional-order memristive or memristor-based neural networks has been gradually carried out (see Chen et al. 2014, 2015; Rakkiyappan et al. 2014, 2015b; Velmurugan and Rakkiyappan 2016; Velmurugan et al. 2016), it is worth noting that fractional-order memristive neural networks can exhibit complicated dynamics or chaotic behaviors if the network’s parameters and time delays are appropriately specified.

Noticed that many static or dynamic control laws have been designed to stabilize nonlinear control systems, for instance, Chandrasekar and Rakkiyappan (2016), Chen et al. (2015), Guo et al. (2013), Huang et al. (2009), Lou et al. (2013), Mathiyalagan et al. (2015), Rakkiyappan et al. (2015a), Wu et al. (2016), Yang and Tong (2016). In allusion to different system structures and actual control requirements, lots of stabilization criteria are established, for example, periodic intermittent stabilization (Huang et al. 2009), robust stabilization (Yang and Tong 2016), finite-time stabilization (Zhang et al. 2016), impulsive stabilization (Chandrasekar and Rakkiyappan 2016; Huang 2010; Lou et al. 2013). Despite these fruitful achievements, some stabilization approaches can hardly be widely applied in practical problems due to high gain. In addition, an undeniable fact is that stabilization control schemes of fractional-order systems is little studied. Hence, it is necessary to investigate some appropriate controllers for stabilization of fractional-order systems.

Inspired by the above discussion, in this article, we will study the global $O(t^{-\alpha })$ stabilization problem for a class of fractional-order memristive neural networks with time delays. We first introduce the concepts about fractional calculation and global stabilization of fractional-order systems. Secondly, for exploring some simple useful controllers, linear state feedback control law and linear output feedback control law are designed to stabilize the fractional-order systems. In addition, stabilization criteria in form of algebraic inequalities are derived by utilizing a new fractional Lyapunov method instead of classical Gronwall inequality. The conditions can be easily verified.

## Fractional calculation and model description

### Fractional calculation concepts

First of all, some basics of fractional calculation are given which will be used in the later.

#### **Definition 1**

(Chen and Chen 2016) The fractional integral with fractional order $\alpha >0$ of function *f*(*t*) is defined as

$$ {}_{t_0}^{RL}D_{t}^{-\alpha }f(t)=\frac{1}{\Gamma (\alpha )}\int _{t_0}^{t}(t-s)^{\alpha -1}f(s){\mathrm {d}}s, $$

where $t\ge t_0$, $\Gamma (\cdot )$ is the Gamma function, that is

$$ \Gamma (\alpha )=\int _0^{\infty }s^{\alpha -1}e^{-s}{\mathrm {d}}s. $$

#### **Definition 2**

(Chen and Chen 2016) The Riemann–Liouville derivative with fractional order $\alpha >0$ of function *f*(*t*) is defined as

$$ {}_{t_0}^{RL}D_{t}^{\alpha }f(t)=\frac{1}{\Gamma (n-\alpha )}\frac{{\mathrm {d}}^n}{{\mathrm {d}}t^n}\int _{t_0}^{t}\frac{f(s)}{(t-s)^{\alpha -n+1}}{\mathrm {d}}s, $$

where $t\ge t_0$, $n-1<\alpha <n$, *n* is a positive integer. Moreover, when $0<\alpha <1$, that is

$$ {}_{t_0}^{RL}D_{t}^{\alpha }f(t)=\frac{1}{\Gamma (1-\alpha )}\frac{{\mathrm {d}}}{{\mathrm {d}}t}\int _{t_0}^{t}\frac{f(s)}{(t-s)^{\alpha }}{\mathrm {d}}s. $$

#### **Definition 3**

(Chen and Chen 2016) The Caputo derivative with fractional order $\alpha >0$ of function $f\in C^{n+1}([t_0,+\infty ),{\mathcal {R}})$ is defined as

$$ {}_{t_0}^{C}D_{t}^{\alpha }f(t)=\frac{1}{\Gamma (n-\alpha )}\int _{t_0}^{t}\frac{f^{(n)}(s)}{(t-s)^{\alpha -n+1}}{\mathrm {d}}s,$$

where $t\ge t_0$, $n-1<\alpha <n$, *n* is a positive integer. Moreover, when $0<\alpha <1$, that is

$$ {}_{t_0}^{C}D_{t}^{\alpha }f(t)=\frac{1}{\Gamma (1-\alpha )}\int _{t_0}^{t}\frac{f^{'}(s)}{(t-s)^{\alpha }}{\mathrm {d}}s.$$

#### **Lemma 1**

(Chen and Chen 2016) *If*$f\in C^1([0,+\infty ),{\mathcal {R}})$, *then the following properties hold*:

1. $${}_{t_0}^{C}D_{t}^{\alpha }f(t)={}_{t_0}^{RL}D_{t}^{\alpha }f(t)-\frac{f(t_0)}{\Gamma (1-\alpha )}(t-t_0)^{-\alpha }$$.
2. *Iff*(*t*) *and*$\vartheta$*and their all derivatives are continuous in*$[t_0,t]$, *then Leibniz rule for fractional differentiation can be expressed as follows:*
  $$ {}_{t_0}^{RL}D_{t}^{\alpha }(\vartheta (t)f(t))=\sum \limits _{k=0}^{n} \frac{{\mathrm {d}}^{k}\vartheta (t)}{{\mathrm {d}}t^k}{\alpha \atopwithdelims ()k}{}_{t_0}^{RL}D_{t}^{\alpha -k}f(t)-R_{n}^{\alpha }, $$

*where*$$0<\alpha <1$$, $$n\ge \alpha $$,

$$\begin{aligned} &R_{n}^{\alpha }(t)=  {} \frac{(-1)^{n}(t-\alpha )^{n-\alpha +1}}{n! \Gamma (-\alpha )} \int _{0}^{1} \int _{0}^{1} F_{\alpha }(t,\xi ,\eta ) {\mathrm {d}} \xi {\mathrm {d}} \eta ,\\ &F_{\alpha }(t,\xi ,\eta )=  {} f(t_{0}+\eta (t-t_0)) \vartheta ^{n+1} (t_0+(t-t_0)(\xi +\eta -\xi \eta )), \end{aligned}$$

*and*

$$ {\alpha \atopwithdelims ()k}=\frac{\Gamma (\alpha +1)}{k!\Gamma (\alpha -k+1)}. $$

### Model description

Consider the fractional-order memristive neural networks with time delays (FMDNNs) described by the following fractional-order equations: for $i=1,2,\ldots ,n,$

1 $$\begin{aligned} {}_{t_0}^{C}D_{t}^{\alpha }x_i(t)&=  {} -x_i(t)+\sum \limits _{j = 1}^n a_{ij}(x_j(t))g_j(x_j(t))\nonumber \\&\quad +\sum \limits _{j = 1}^n b_{ij}(x_j(t))f_j(x_j(t-\tau (t))) + u_i(t), \end{aligned}$$

where $0<\alpha <1$, *n* is the number of neurons in the networks, $x_i(t)$ is the state variable of the *i*th neuron, $g_j(\cdot )$, $f_j(\cdot )$ denotes the output of the *j*th unit at time *t* and $t-\tau (t)$, respectively, and $g_j(0)=f_j(0)=0$. $\tau (t)$ corresponds to the transmission delay at time *t* and $0\le \tau (t)\le \tau$. $u_i(t)$ denotes the external input, $a_{ij}(x_j(t))$ and $b_{ij}(x_j(t))$ represent memristive weights, which are defined as:

2 $$\begin{aligned} a_{ij}(x_j(t))=\left\{ \begin{array}{ll} {\hat{a}}_{ij}, &{}\quad \left| x_j(t)\right|>T_j, \\ {\check{a}}_{ij}, &{}\quad \left| x_j(t)\right|<T_j, \end{array}\right. \quad b_{ij}(x_j(t))=\left\{ \begin{array}{ll} {\hat{b}}_{ij}, &{}\quad \left| x_j(t)\right| >T_j, \\ {\check{b}}_{ij}, &{}\quad \left| x_j(t)\right| <T_j, \end{array}\right. \end{aligned}$$

for $$i,j=1,2,\ldots ,n$$, $$a_{ij}$$(±*T*$$_{j}$$) = $${\hat{a}}_{ij}$$ or $${\check{a}}_{ij},$$$$b_{ij}$$(±*T*$$_{j}$$) = $${\hat{b}}_{ij}$$ or $${\check{b}}_{ij},$$ where the switching jumps $$T_j> 0$$, $${\hat{a}}_{ij}$$, $${\check{a}}_{ij}$$, $${\hat{b}}_{ij}$$, and $${\check{b}}_{ij}$$ are constants.

#### *Remark 1*

Note that $a_{ij}(x_j(t))$ and $b_{ij}(x_j(t))$ are discontinuous in system (1), then the classical definition of solution for differential equations cannot be applied to (1). To deal with this issue, we introduce the concept of Filippov solution.

#### **Definition 4**

(Rakkiyappan et al. 2014) For system ${}_{t_0}^{C}D_{t}^{\alpha }x(t)=g(x)$, $0<\alpha <1$, $x\in {\mathcal{R}}^n$, with a discontinuous right-hand side, a set-valued map is defined as

$$ \psi (x)=\bigcap \limits _{\delta >0} \bigcap \limits _{\mu (N)=0} co\left[ g(B(x,\delta ){\backslash}N)\right] , $$

where *co*[*E*] is the closure of convex hull of set *E*, $B(x,\delta )=\{y:\Vert y-x \Vert \le \delta \}$, and $\mu (N)$ is a Lebesgue measure of set *N*. If *x*(*t*), $t\in [t_0,T]$, is called the solution in Filippov sense of the Cauchy problem for system ${}_{t_0}^{C}D_{t}^{\alpha }x(t)=g(x)$, $0<\alpha <1$, $x\in {\mathcal{R}}^n$, with initial condition $x(t_0)=x_0$, when it is absolutely continuous, and satisfies the differential inclusion as follows:

$$ {}_{t_0}^{C}D_{t}^{\alpha }x(t) \in \psi (x),\quad for\quad a.e. \quad t\in [t_0,T]. $$

For FMDNNs (1), define the set-value maps

$$\begin{aligned}&K(a_{ij}(x_j(t)))=\left\{ \begin{array}{ll} \hat{a}_{ij}, &{}\quad \left| x_j(t)\right|>T_j, \\ co\left\{ \hat{a}_{ij},\check{a}_{ij}\right\} , &{}\quad \left| x_j(t)\right| =T_j, \\ {\check{a}}_{ij}, &{}\quad \left| x_j(t)\right|<T_j, \end{array}\right. \\&K(b_{ij}(x_j(t)))=\left\{ \begin{array}{ll} {\hat{b}}_{ij}, &{}\quad \left| x_j(t)\right| >T_j, \\ co\left\{ {\hat{b}}_{ij},{\check{b}}_{ij}\right\} , &{}\quad \left| x_j(t)\right| =T_j, \\ {\check{b}}_{ij}, &{}\quad \left| x_j(t)\right| <T_j, \end{array}\right. \end{aligned}$$

for $i,j=1,2,\ldots ,n$, where $co\{{\hat{a}}_{ij},{\check{a}}_{ij}\}$ denotes the closure of convex hull generated by real numbers ${\hat{a}}_{ij}$ and ${\check{a}}_{ij}$, $co\{{\hat{b}}_{ij},{\check{b}}_{ij}\}$ denotes the closure of convex hull generated by real numbers ${\hat{b}}_{ij}$ and ${\check{b}}_{ij}$.

Throughout this article we denote $a_{ij}^{m}=\max \nolimits _{1\le i,j \le n}\{|{\hat{a}}_{ij}|,|{\check{a}}_{ij}|\}$, $b_{ij}^{m}=\max \nolimits _{1\le i,j \le n}\{|{\hat{b}}_{ij}|,|{\check{b}}_{ij}|\}$. For *n*-dimensional vector $v=(v_{1},v_{2},\ldots ,v_{n})^{T}$, the norm of vector *v* is recorded as $\Vert v\Vert =\sum \nolimits _{i=1}^{n} |v_{i}|$. $C_{\tau }:=C([-\tau ,0],{\mathcal {R}})$ is a Banach space of all continuous functions $\varphi :[-\tau ,0]\rightarrow {\mathcal {R}}$. For $\varphi \in C_{\tau }$, let $\Vert \varphi \Vert _{C}=\sup \nolimits _{s\in [-\tau ,0]} \Vert \varphi (s)\Vert$.

Throughout this article, let us suppose: the activation functions $g_i$, $f_i$, $i=1,2,\ldots ,n$, are global Lipschitz, that is, for all $u,v \in {\mathcal {R}},$ there exist positive constants $G_i$, $F_i$ such that

$$ \left| g_i(u)-g_{i}(v)\right| \le G_i \left| u-v\right| ,\quad \left| f_i(u)-f_{i}(v)\right| \le F_i \left| u-v\right| . $$

The objective of this article is to investigate the global $$O(t^{-\alpha })$$ stabilization problem for system (1). Therefore, the stabilization problem will be converted to find the suit controller $$u_i(t)$$$$(i=1,2,\ldots ,n)$$ such that zero solution of the closed-loop system of (1) is globally $$O(t^{-\alpha })$$ stable.

From the theories of differential inclusions and set-valued maps, the Filippov solution of FMDNNs (1) can be defined in the following form.

#### **Definition 5**

A function $x(t)=(x_1(t), x_2(t), \ldots , x_n(t))^{T}$ is said to be a Filippov solution of FMDNNs (1) on [0, *T*) with initial conditions $x(s)=\varphi (s)$, $s\in [-\tau ,0]$, if *x*(*t*) is absolutely continuous on any compact interval of [0, *T*), and

3 $$\begin{aligned}&{}_{t_0}^{C}D_{t}^{\alpha }x_i(t)\in -x_i(t)+\sum \limits _{j = 1}^n K(a_{ij}(x_j(t)))g_j(x_j(t))\nonumber \\&\quad \qquad +\,\sum \limits _{j = 1}^n K(b_{ij}(x_j(t)))f_j(x_j(t-\tau (t))) + u_i(t), \end{aligned}$$

for $t\ge t_0$, $i=1,2,\ldots ,n$. Or equivalently, for $i,j=1,2,\ldots ,n$, there exist $\gamma _{ij}^{a}(x_j(t))\in K(a_{ij}(x_j(t)))$, $\gamma _{ij}^{b}(x_j(t))\in K(b_{ij}(x_j(t)))$ such that

4 $$\begin{aligned} {}_{t_0}^{C}D_{t}^{\alpha }x_i(t)&=  -x_i(t)+\sum \limits _{j = 1}^n \gamma _{ij}^{a}(x_j(t))g_j(x_j(t))\nonumber \\&\quad +\sum \limits _{j = 1}^n \gamma _{ij}^{b}(x_j(t))f_j(x_j(t-\tau (t))) + u_i(t). \end{aligned}$$

#### *Remark 2*

Based on the definitions of Filippov solution and fractional-order differential inclusion, we know that FMDNNs (1) is equivalent to the fractional-order differential inclusion (3) in the Filippov framework.

Next, definitions of global $O(t^{-\alpha })$ stability and global $O(t^{-\alpha })$ stabilization are given.

#### **Definition 6**

[*Global*$O(t^{-\alpha })$*stability*] The zero solution of FMDNNs (1), where $u_i(t)=0$, is said to be globally $O(t^{-\alpha })$ stable if there exists a positive constant *M* such that $\Vert x(t,t_0,\varphi )\Vert \le M \Vert \varphi \Vert _{C} O(t^{-\alpha })$ for any $\varphi \in C_{\tau }$ and $t \ge t_0$.

#### **Definition 7**

[*Global*$O(t^{-\alpha })$*stabilization*] FMDNNs (1) is said to be globally $O(t^{-\alpha })$ stabilized if there exists an appropriate feedback control law such that the closed-loop system of (1) is globally $O(t^{-\alpha })$ stable.

## Main results

### State feedback control law

Two kinds of linear controller about state feedback are given, i.e., the linear controller without or with time delays. Firstly, we propose the following state control rule without time delays:

5 $$ u_i(t)=\sum \limits _{j = 1}^n p_{ij}x_j(t), $$

for $$i=1,2,\ldots ,n$$.

#### **Theorem 1**

*FMDNNs* (1) *with the state feedback control rule* (5) *can be achieved global*$$O(t^{-\alpha })$$*stabilization for any*$$\varphi \in C_{\tau }$$*if there exist a constant*$$r>\tau $$*andnpositive constants*$$\beta _i$$$$(i=1,2,\ldots ,n)$$*such that*

6 $$ \sum \limits _{j = 1}^n \beta _j p_{ij} \le \beta _i\left( 1-\frac{1+\alpha }{r^{\alpha }\Gamma (2-\alpha )}\right) -\sum \limits _{j = 1}^n \beta _j \left( a_{ij}^{m}G_j+\left( \frac{r}{r-\tau }\right) ^{\alpha }b_{ij}^{m}F_j\right) , $$

*for all*$$i=1,2,\ldots ,n$$.

#### *Proof*

Define two Lyapunov functions as follows:

7 $$\begin{aligned} \left\{ \begin{array}{l} W(t)=\max \left\{ \frac{|x_i(t)|}{\beta _i},i=1,2,\ldots ,n \right\} ,\\ V(t)=(t-t_0+r)^\alpha W(t), \end{array} \right. \end{aligned}$$

and let

8 $$\begin{aligned} \left\{ \begin{array}{l} {\overline{W}}(t)=\sup \nolimits _{-\tau \le \theta \le t} W(\theta ), \\ {\overline{V}}(t)=\sup \nolimits _{-\tau \le \theta \le t} V(\theta ) , \end{array} \right. \end{aligned}$$

for $$t\ge t_0$$.

From Leibniz rule for fractional differentiation, we have

9 $$\begin{aligned} {}_{t_0}^{C}D_{t}^{\alpha } V(t)&=  {} {}_{t_0}^{RL}D_{t}^{\alpha }V(t)-\frac{V(t_0)}{\Gamma (1-\alpha )}(t-t_0)^{-\alpha }\nonumber \\&=  {} {}_{t_0}^{RL}D_{t}^{\alpha }\left( (t-t_{0}+r)^{\alpha }W(t)\right) -\frac{r^{\alpha }W(t_0)}{\Gamma (1-\alpha )}(t-t_0)^{-\alpha }\nonumber \\&= {} (t-t_{0}+r)^{\alpha }{}_{t_0}^{RL}D_{t}^{\alpha }W(t) +\dfrac{\Gamma (\alpha +1)}{\Gamma (\alpha )}\alpha (t-t_{0}+r)^{\alpha -1}{}_{t_0}^{RL}D_{t}^{\alpha -1}W(t)\nonumber \\&\quad +\dfrac{\Gamma (\alpha +1)}{2 \Gamma (\alpha -1)}\alpha (\alpha -1)(t-t_{0}+r)^{\alpha -2}{}_{t_0}^{RL}D_{t}^{\alpha -2}W(t)\nonumber \\&\quad -R_2^{\alpha }(t)-\dfrac{r^{\alpha }W(t_0)}{\Gamma (1-\alpha )}(t-t_0)^{-\alpha }\nonumber \\&\le  {} (t-t_{0}+r)^{\alpha }{}_{t_0}^{C}D_{t}^{\alpha }W(t) +(t-t_{0}+r)^{\alpha } \frac{W(t_0)}{\Gamma (1-\alpha )} (t-t_0)^{-\alpha }\nonumber \\&\quad +\alpha ^2(t-t_{0}+r)^{\alpha -1}{}_{t_0}^{RL}D_{t}^{\alpha -1}W(t)\nonumber \\&\quad +\frac{\alpha ^2(\alpha -1)^2}{2}(t-t_{0}+r)^{\alpha -2}{}_{t_0}^{RL}D_{t}^{\alpha -2}W(t) -\frac{r^{\alpha }W(t_0)}{\Gamma (1-\alpha )}(t-t_0)^{-\alpha }\nonumber \\&\le {} (t-t_{0}+r)^{\alpha }{}_{t_0}^{C}D_{t}^{\alpha }W(t) +\left[ \left( \frac{t-t_{0}+r}{t-t_0}\right) ^{\alpha }- \left( \frac{r}{t-t_0}\right) ^{\alpha } \right] \frac{W(t_0)}{\Gamma (1-\alpha )}\nonumber \\&\quad +\alpha ^2(t-t_{0}+r)^{\alpha -1}{}_{t_0}^{RL}D_{t}^{\alpha -1}W(t)\nonumber \\&\quad +\frac{\alpha ^2(\alpha -1)^2}{2}(t-t_{0}+r)^{\alpha -2}{}_{t_0}^{RL}D_{t}^{\alpha -2}W(t)\nonumber \\ &\le {} (t-t_{0}+r)^{\alpha }{}_{t_0}^{C}D_{t}^{\alpha }W(t) +(1+2 \alpha )\frac{{\overline{V}}(t)}{r^{\alpha } \Gamma (1-\alpha )}\nonumber \\&\quad +\alpha ^2(t-t_{0}+r)^{\alpha -1}{}_{t_0}^{RL}D_{t}^{\alpha -1}W(t)\nonumber \\&\quad +\frac{\alpha ^2(\alpha -1)^2}{2}(t-t_{0}+r)^{\alpha -2}{}_{t_0}^{RL}D_{t}^{\alpha -2}W(t), \end{aligned}$$

for $$t\ge t_{0}$$.

By computing, it follows that

10 $$\begin{aligned} \alpha ^2(t-t_{0}+r)^{\alpha -1}{}_{t_0}^{RL}D_{t}^{\alpha -1}W(t)&=\alpha ^2(t-t_{0}+r)^{-(1-\alpha )}{}_{t_0}^{RL}D_{t}^{-(1-\alpha )}W(t)\nonumber \\&\le \alpha ^2(t-t_{0}+r)^{-(1-\alpha )}\frac{1}{\Gamma (1-\alpha )}\overline{W}(t)\int _{t_{0}}^t (t-s)^{-\alpha } {\mathrm {d}}s\nonumber \\&\le \alpha ^2(t-t_{0}+r)^{-(1-\alpha )}\frac{(t-t_0)^{1-\alpha }}{(1-\alpha )\Gamma (1-\alpha )}\overline{W}(t)\nonumber \\&\le \alpha ^2\left( \frac{t-t_0}{t-t_{0}+r}\right) ^{1-\alpha }\frac{1}{\Gamma (2-\alpha )}\overline{W}(t)\nonumber \\&\le \frac{\alpha ^2}{\Gamma (2-\alpha )}W({\widetilde{\theta }})\nonumber \\&\le \frac{\alpha ^2}{r^{\alpha }\Gamma (2-\alpha )}{\overline{V}}(t), \end{aligned}$$

and

11 $$\begin{aligned}&\frac{\alpha ^2(\alpha -1)^2}{2}(t-t_{0}+r)^{\alpha -2}{}_{t_0}^{RL}D_{t}^{\alpha -2}W(t)\nonumber \\&\quad \le \frac{\alpha ^2(\alpha -1)^2}{2}(t-t_{0}+r)^{\alpha -2}\frac{1}{\Gamma (2-\alpha )}\int _{t_{0}}^t (t-s)^{1-\alpha }W(s){\mathrm {d}}s\nonumber \\&\quad \le \frac{\alpha ^2(\alpha -1)^2}{2}(t-t_{0}+r)^{\alpha -2}\frac{\overline{W}(t)}{\Gamma (2-\alpha )}\int _{t_{0}}^t (t-s)^{1-\alpha }{\mathrm {d}}s\nonumber \\&\quad \le \frac{\alpha ^2(\alpha -1)^2}{2}(t-t_{0}+r)^{\alpha -2}\frac{(t-t_0)^{2-\alpha }\overline{W}(t)}{(2-\alpha )\Gamma (2-\alpha )}\nonumber \\&\quad \le \frac{\alpha ^2(\alpha -1)^2}{2}\left( \frac{t-t_0}{t-t_{0}+r}\right) ^{2-\alpha }\frac{\overline{W}(t)}{(2-\alpha )\Gamma (2-\alpha )}\nonumber \\&\quad \le \frac{\alpha ^2(\alpha -1)^2}{2}\frac{\overline{W}(t)}{(2-\alpha )\Gamma (2-\alpha )}\nonumber \\&\quad \le \frac{\alpha ^2(\alpha -1)^2}{2r^{\alpha }(2-\alpha )\Gamma (2-\alpha )}{\overline{V}}(t)\nonumber \\&\quad \le \frac{\alpha ^2}{r^{\alpha }\Gamma (2-\alpha )}{\overline{V}}(t), \end{aligned}$$

for $$t\ge t_{0}$$, $${\overline{W}}(t)=W({\widetilde{\theta }})$$, where $${\widetilde{\theta }}\in [-\tau ,t]$$.

From (9) to (11), we have

12 $$\begin{aligned} {}_{t_0}^{C}D_{t}^{\alpha }V(t)&\le (t-t_{0}+r)^{\alpha } {}_{t_0}^{C}D_t^{\alpha } W(t)+\frac{ 2 \alpha ^2}{r^{\alpha }\Gamma (2-\alpha )}{\overline{V}}(t)+ \frac{(1+2\alpha ) {\overline{V}}(t)}{r^{\alpha } \Gamma (1-\alpha )}\nonumber \\&= (t-t_{0}+r)^{\alpha } {}_{t_0}^{C}D_t^{\alpha } W(t)+\frac{1+\alpha }{r^{\alpha }\Gamma (2-\alpha )}{\overline{V}}(t). \end{aligned}$$

It is obvious that there exists a $k\in \{1,2,\ldots ,n\}$ such that

$$ W(t)=\frac{|x_k(t)|}{\beta _k}, $$

for given $$t\ge t_0$$.

From (7) and (8), we have

13 $$\begin{aligned} {}_{t_0}^{C}D_{t}^{\alpha } W(t)&=\frac{1}{\beta _k}{}_{t_0}^{C}D_{t}^{\alpha }|x_k(t)|\nonumber \\&\le \frac{1}{\beta _k} sgn(x_k(t)) {}_{t_0}^{C}D_{t}^{\alpha }x_k(t)\nonumber \\&\le \frac{1}{\beta _k} sgn(x_k(t))\left\{ -x_k(t)+\sum \limits _{j=1}^{n}\gamma _{kj}^{a}(x_j(t))g_j(x_j(t))\right. \nonumber \\&\quad +\left. \sum \limits _{j=1}^{n}\gamma _{kj}^{b}(x_j(t))f_j(x_j(t-\tau (t)))+\sum \limits _{j=1}^{n}p_{kj}x_j(t)\right\} \nonumber \\&\le \frac{1}{\beta _k}sgn(x_k(t))\left\{ -x_k(t)+\sum \limits _{j=1}^{n} a_{kj}^{m}G_j |x_j(t)|\right. \nonumber \\&\quad +\left. \sum \limits _{j=1}^{n} b_{kj}^{m}F_j|x_j(t-\tau (t))|+\sum \limits _{j=1}^{n}p_{kj}x_j(t)\right\} \nonumber \\&\le - \frac{|x_k(t)|}{\beta _k}+\frac{1}{\beta _k}\sum \limits _{j=1}^{n} \beta _j a_{kj}^{m} G_j \frac{|x_j(t)|}{\beta _j} +\frac{1}{\beta _k} \sum \limits _{j=1}^{n} \beta _j b_{kj}^{m} F_j \frac{|x_j(t-\tau (t))|}{\beta _j}\nonumber \\&\quad +\frac{1}{\beta _k} \sum \limits _{j=1}^{n} \beta _j p_{kj} \frac{|x_j(t)|}{\beta _j}\nonumber \\&\le -W(t)+\frac{1}{\beta _k}\sum \limits _{j=1}^n \beta _j (a_{kj}^{m}G_j+p_{kj} )W(t) +\frac{1}{\beta _k}\sum \limits _{j=1}^{n} \beta _j b_{kj}^{m} F_j W(t-\tau (t))\nonumber \\&\le -\left[ 1-\frac{1}{\beta _k}\sum \limits _{j=1}^n \beta _j (a_{kj}^{m}G_j+p_{kj})\right] W(t) +\frac{1}{\beta _k}\sum \limits _{j=1}^{n} \beta _j b_{kj}^{m} F_j \overline{W}(t-\tau (t)). \end{aligned}$$

And hence

14 $$\begin{aligned} {}&{}(t-t_0+r)^{\alpha }{}_{t_0}^{C}D_{t}^{\alpha } W(t)\nonumber \\&\quad \le -(t-t_0+r)^{\alpha }\left[ 1-\frac{1}{\beta _k}\sum \limits _{j=1}^n \beta _j \left( a_{kj}^{m}G_j+p_{kj}\right) \right] W(t)\nonumber \\&\quad \qquad +(t-t_0+r)^{\alpha }\frac{1}{\beta _k}\sum \limits _{j=1}^{n} \beta _j b_{kj}^{m} F_j {\overline{W}}(t-\tau (t))\nonumber \\&\quad \le -\left[ 1-\frac{1}{\beta _k}\sum \limits _{j=1}^n \beta _j \left( a_{kj}^{m}G_j+p_{kj}\right) \right] V(t) +\frac{(t-t_0+r)^{\alpha }}{\beta _k\left( t-t_0+r+{\overline{\theta }}\right) ^{\alpha }}\sum \limits _{j=1}^{n} \beta _j b_{kj}^{m} F_j {\overline{V}}(t)\nonumber \\&\quad \le -\left[ 1-\sum \limits _{j=1}^n \frac{\beta _j}{\beta _k} (a_{kj}^{m}G_j+p_{kj})-\left( \frac{r}{r+\overline{\theta }}\right) ^{\alpha }\sum \limits _{j=1}^{n}\frac{\beta _j}{\beta _k} b_{kj}^{m} F_j\right] V(t)\nonumber \\&\quad \le -\left[ 1-\sum \limits _{j=1}^n \frac{\beta _j}{\beta _k} \left( a_{kj}^{m}G_j+p_{kj}\right) -\left( \frac{r}{r-\tau }\right) ^{\alpha }\sum \limits _{j=1}^{n}\frac{\beta _j}{\beta _k} b_{kj}^{m} F_j\right] V(t), \end{aligned}$$

where ${\overline{\theta}}\in [-\tau ,t]$ such that ${\overline{V}}(t)=(t-t_0+{\overline{\theta }}+r)^{\alpha } {\overline{W}}(t)$, when ${\overline{V}}(t)=V(t)$.

From (12) and (14), we have

15 $$\begin{aligned} {}_{t_0}^{C}D_{t}^{\alpha }V(t)&\le -\left[ 1-\sum \limits _{j=1}^n \frac{\beta _j}{\beta _k} (a_{kj}^{m}G_j+p_{kj})-\left( \frac{r}{r-\tau }\right) ^{\alpha }\sum \limits _{j=1}^{n}\frac{\beta _j}{\beta _k} b_{kj}^{m} F_j\right] V(t) +\frac{1+ \alpha }{r^{\alpha } \Gamma (2-\alpha )}{\overline{V}}(t)\nonumber \\&\le \left\{ -\left[ 1-\sum \limits _{j=1}^n \frac{\beta _j}{\beta _k} \left( a_{kj}^{m}G_j+p_{kj}\right) -\left( \frac{r}{r-\tau }\right) ^{\alpha }\sum \limits _{j=1}^{n}\frac{\beta _j}{\beta _k} b_{kj}^{m} F_j\right] +\frac{1+ \alpha }{r^{\alpha } \Gamma (2-\alpha )}\right\} V(t), \end{aligned}$$

when $$\overline{V}(t)=V(t)$$.

From (6), it follows that

16 $$ {}_{t_0}^{C}D_{t}^{\alpha } {\overline{V}}(t)\le 0, $$

for all $$t\ge t_0$$.

On the basis of Definition 2 and Lemma 1, the following inequality holds

$$ \frac{1}{\Gamma (1-\alpha )}\frac{{\mathrm {d}}}{{\mathrm {d}}t} \int _{t_0}^{t} \frac{{\overline{V}}(s)}{(t-s)^{\alpha }} {\mathrm {d}}s \le \frac{{\overline{V}}(t_0)}{\Gamma (1-\alpha )}(t-t_0)^{-\alpha }. $$

It yields

17 $$ {\overline{V}}(t)\le {\overline{V}}(t_0), $$

for $t\ge t_0$. Hence for $i=1,2,\ldots ,n,$

18 $$\begin{aligned} |x_i(t)|&\le \beta _i W(t)\nonumber \\&=\beta _i \frac{V(t)}{(t-t_0+r)^{\alpha }}\le \beta _i \frac{{\overline{V}}(t)}{(t-t_0+r)^{\alpha }}\nonumber \\&\le \beta _i \frac{V(t_0)}{(t-t_0+r)^{\alpha }}=\beta _i \frac{r^{\alpha } {\overline{W}}(t_0)}{(t-t_0+r)^{\alpha }}\nonumber \\&\le \frac{\beta _i r^{\alpha } \Vert \varphi \Vert _C}{\beta _{\min }(t-t_0+r)^{\alpha }}, \end{aligned}$$

where $\beta _{\min }=\min \{\beta _i, i=1,2,\ldots ,n\}$, for $t\ge t_0$, which implies

$$ \Vert x(t)\Vert \le \frac{\Theta r^{\alpha }\Vert \varphi \Vert _C}{(t-t_0+r)^{\alpha }}, $$

where $\Theta =\frac{1}{\beta _{\min }}\sum \nolimits _{i=1}^{n}\beta _i$. Therefore, FMDNNs (1) can be achieved global $O(t^{-\alpha })$ stabilization under the designed control law (5). $\square$

In the following, we propose the following state control rule with time delays:

19 $$ u_i(t)=\sum \limits _{j=1}^{n}p_{ij}x_j(t)+\sum \limits _{j=1}^{n}q_{ij}x_{j}(t-\tau (t)),$$

for $$i=1,2,\ldots ,n$$.

#### **Theorem 2**

*FMDNNs* (1) *with the state feedback control rule* (19) *can be achieved global*$$O(t^{-\alpha })$$*stabilization for any*$$\varphi \in C_{\tau }$$*if there exist a constant*$$r>\tau $$*andnpositive constants*$$\beta _i$$$$(i=1,2,\ldots ,n)$$*such that*

20 $$ \sum \limits _{j=1}^{n} \beta _j \left( p_{ij}+\left( \frac{r}{r-\tau }\right) ^{\alpha }q_{ij}\right)\le {} \beta _i \left( 1-\frac{1+\alpha }{r^{\alpha }\Gamma (2-\alpha )}\right)  -\sum \limits _{j=1}^{n} \beta _j \left( a_{ij}^{m}G_j+\left( \frac{r}{r-\tau }\right) ^{\alpha }b_{ij}^{m}F_j\right) ,$$

*for all*$$i=1,2,\ldots ,n$$.

#### *Proof*

Define two Lyapunov functions as follows:

21 $$\begin{aligned} \left\{ \begin{array}{l} W(t)=\max \left\{ \frac{|x_i(t)|}{\beta _i},i=1,2,\ldots ,n \right\} ,\\ V(t)=(t-t_0+r)^\alpha W(t), \end{array} \right. \end{aligned}$$

and let

22 $$\begin{aligned} \left\{ \begin{array}{l} {\overline{W}}(t)=\sup \nolimits _{-\tau \le \theta \le t} W(\theta ), \\ {\overline{V}}(t)=\sup \nolimits _{-\tau \le \theta \le t} V(\theta ), \end{array} \right. \end{aligned}$$

for $$t\ge t_0$$.

Through Theorem 1, we have

23 $$ {}_{t_0}^{C}D_{t}^{\alpha } V(t) \le (t-t_0+r)^{\alpha }{}_{t_0}^{C}D_{t}^{\alpha }W(t)+\frac{1+\alpha }{r^{\alpha }\Gamma (2-\alpha )}{\overline{V}}(t). $$

It is obvious that there exists a $k\in \{1,2,\ldots ,n\}$ such that

$$ W(t)=\frac{|x_k(t)|}{\beta _k}, $$

for given $$t\ge t_0$$.

From (21) and (22), we have

24 $$\begin{aligned} {}_{t_0}^{C}D_{t}^{\alpha }W(t)&\le \frac{1}{\beta _k}sgn(x_k(t)){}_{t_0}^{C}D_{t}^{\alpha }x_k(t)\nonumber \\&\le \frac{1}{\beta _k}sgn(x_k(t))\left\{ -x_k(t)+\sum \limits _{j=1}^{n} \gamma _{kj}^{a}(x_j(t))g_j(x_j(t)) + \sum \limits _{j=1}^{n} \gamma _{kj}^{b}(x_j(t))f_j(x_j(t-\tau (t)))\right.\nonumber \\&\quad \left. + \sum \limits _{j=1}^{n}p_{kj}x_j(t)+\sum \limits _{j=1}^{n}q_{kj}x_j(t-\tau (t))\right\} \nonumber \\&\le \frac{1}{\beta _k}sgn(x_k(t))\left\{ -x_k(t)+\sum \limits _{j=1}^{n} a_{kj}^{m} G_j|x_j(t)| +\sum \limits _{j=1}^{n} b_{kj}^{m} F_j|x_j(t-\tau (t))|\right.\nonumber \\&\quad + \left. \sum \limits _{j=1}^{n}p_{kj}x_j(t)+\sum \limits _{j=1}^{n}q_{kj}x_j(t-\tau (t))\right\} \nonumber \\&\le -\left[ 1-\frac{1}{\beta _k}\sum \limits _{j=1}^{n}\beta _j (a_{kj}^{m}G_j+p_{kj})\right] W(t) +\frac{1}{\beta _k}\sum \limits _{j=1}^{n}\beta _j\left(b_{kj}^{m}F_j+q_{kj}\right) {\overline{W}}(t). \end{aligned}$$

Hence

25 $$ (t-t_0+r)^{\alpha }{}_{t_0}^{C}D_{t}^{\alpha }W(t)\le {} -\left[ 1-\sum \limits _{j=1}^{n} \frac{\beta _j}{\beta _k}\left( a_{kj}^{m}G_j+p_{kj} +\left( \frac{r}{r-\tau }\right) ^{\alpha }\left( b_{kj}^{m}F_j+q_{kj}\right) \right) \right] V(t), $$

where ${\overline{\theta}}\in [-\tau ,t]$ such that ${\overline{V}}(t)=(t+{\overline{\theta}}-t_0+r)^{\alpha } {\overline{W}}(t)$, when ${\overline{V}}(t)=V(t)$.

From (23) and (25), we have

26 $$\begin{aligned} {}_{t_0}^{C}D_{t}^{\alpha } V(t)&\le -\left[ 1-\sum \limits _{j=1}^{n}\frac{\beta _j}{\beta _k}\left( a_{kj}^{m}G_j+p_{kj}+ \left( \frac{r}{r-\tau }\right) ^{\alpha }\left( b_{kj}^{m}F_j+q_{kj}\right) \right) \right] V(t) +\frac{1+\alpha }{r^{\alpha }\Gamma (2-\alpha )} {\overline{V}}(t) \nonumber \\&\le \left\{ -\left[ 1-\sum \limits _{j=1}^{n}\frac{\beta _j}{\beta _k}\left( a_{kj}^{m}G_j+p_{kj}+ \left( \frac{r}{r-\tau }\right) ^{\alpha }\left( b_{kj}^{m}F_j+q_{kj}\right) \right) \right] +\frac{1+\alpha }{r^{\alpha }\Gamma (2-\alpha )}\right\} V(t), \end{aligned}$$

when $${\overline{V}}(t)=V(t)$$.

From (20), it follows that

27 $$ {}_{t_0}^{C}D_{t}^{\alpha } {\overline{V}}(t)\le 0, $$

for all $$t \ge t_0$$.

On the basis of Definition 3, we get

28 $$ {\overline{V}}(t)\le {\overline{V}}(t_0), $$

for $t\ge t_0$. Hence for $i=1,2,\ldots ,n,$

29 $$ |x_i(t)|\le \beta _i W(t)\le \frac{\beta _i r^{\alpha }\Vert \varphi \Vert _C}{\beta _{\min }(t-t_0+r)^{\alpha }}, $$

where $\beta _{\min }=\min \{\beta _i, i=1,2,\ldots ,n\}$, for $t\ge t_0,$ it follows

$$ \Vert x(t)\Vert \le \frac{\Theta r^{\alpha }\Vert \varphi \Vert _C}{(t-t_0+r)^{\alpha }}, $$

where $\Theta =\frac{1}{\beta _{\min }}\sum \nolimits _{i=1}^{n}\beta _i$. Therefore, FMDNNs can be achieved global $O(t^{-\alpha })$ stabilization under the designed control law (19). $\square$

### Output feedback control law

Two kinds of linear controller about output feedback are given, i.e., the linear output feedback controller without or with time delays. Firstly, we propose the following output feedback control rule without time delays:

30 $$ u_i(t)=\sum \limits _{j=1}^{n}\omega _{ij}g_j(x_j(t)), $$

for $$i=1,2,\ldots ,n$$.

#### **Theorem 3**

*FMDNNs* (1) *with the output feedback control rule* (30) *can be achieved global*$$O(t^{-\alpha })$$*stabilizationfor any*$$\varphi \in C_{\tau }$$*if there exist a constant*$$r>\tau $$*andnpositive constants*$$\beta _i$$$$(i=1,2,\ldots ,n)$$*such that*

31 $$ \sum \limits _{j = 1}^n \beta _j G_j \omega _{ij}\le {} \beta _i \left( 1-\frac{1+\alpha }{r^{\alpha }\Gamma (2-\alpha )}\right) -\sum \limits _{j = 1}^n \beta _j \left( a_{ij}^{m}G_j+\left( \frac{r}{r-{\tau }}\right) ^{\alpha }b_{ij}^{m}F_j\right) , $$

*for all*$$i=1,2,\ldots ,n$$.

#### *Proof*

Define two Lyapunov functions as follows:

32 $$\begin{aligned} \left\{ \begin{array}{l} W(t)=\max \left\{ \frac{|x_i(t)|}{\beta _i},i=1,2,\ldots ,n \right\} ,\\ V(t)=(t-t_0+r)^\alpha W(t), \end{array} \right. \end{aligned}$$

and let

33 $$\begin{aligned} \left\{ \begin{array}{l} {\overline{W}}(t)=\sup \nolimits _{-\tau \le \theta \le t} W(\theta ), \\ {\overline{V}}(t)=\sup \nolimits _{-\tau \le \theta \le t} V(\theta ), \end{array} \right. \end{aligned}$$

for $$t\ge t_0$$.

Through Theorem 1, we have

34 $$ {}_{t_0}^{C}D_{t}^{\alpha }V(t) \le (t-t_{0}+r)^{\alpha } {}_{t_0}^{C}D_t^{\alpha } W(t)+\frac{1+\alpha }{r^{\alpha }\Gamma (2-\alpha )}{\overline{V}}(t). $$

It is obvious that there exists a $k\in \{1,2,\ldots ,n\}$ such that

$$ W(t)=\frac{|x_k(t)|}{\beta _k}, $$

for given $$t\ge t_0$$.

From (32) and (33), we have

35 $$\begin{aligned} {}_{t_0}^{C}D_{t}^{\alpha } W(t)&=\frac{1}{\beta _k}{}_{t_0}^{C}D_{t}^{\alpha }|x_k(t)|\nonumber \\&\le \frac{1}{\beta _k} sgn(x_k(t)) {}_{t_0}^{C}D_{t}^{\alpha }x_k(t)\nonumber \\&\le \frac{1}{\beta _k} sgn(x_k(t))\left\{ -x_k(t)+\sum \limits _{j=1}^{n}\gamma _{kj}^{a}(x_j(t))g_j(x_j(t))+\sum \limits _{j=1}^{n}\gamma _{kj}^{b}(x_j(t))f_j(x_j(t-\tau (t)))\right. \nonumber \\&\quad +\left. \sum \limits _{j=1}^{n}\omega _{kj} g_j(x_j(t))\right\} \nonumber \\&\le -\frac{|x_k(t)|}{\beta _k}+\frac{1}{\beta _k}\sum \limits _{j=1}^{n}\beta _{j} a_{kj}^{m}G_j \frac{|x_j(t)|}{\beta _j}+\frac{1}{\beta _k}\sum \limits _{j=1}^{n} \beta _{j}b_{kj}^{m}F_j\frac{|x_j(t-\tau (t))|}{\beta _j}\nonumber \\&\quad +\frac{1}{\beta _k}\sum \limits _{j=1}^{n}\beta _{j} \omega _{kj}G_j \frac{|x_j(t)|}{\beta _j}\nonumber \\&\le -\left[ 1-\frac{1}{\beta _k}\sum \limits _{j=1}^n \beta _{j} G_j\left( a_{kj}^{m}+\omega _{kj}\right) \right] W(t) +\frac{1}{\beta _k}\sum \limits _{j=1}^{n} \beta _j b_{kj}^{m} F_j {\overline{W}}(t-\tau (t)). \end{aligned}$$

And hence

36 $$ (t-t_0+r)^{\alpha }{}_{t_0}^{C}D_{t}^{\alpha } W(t)\le {} -\left[ 1-\sum \limits _{j=1}^{n} \frac{\beta _{j}}{\beta _k} \left( G_j\left( a_{kj}^{m}+\omega _{kj}\right)  +\left( \frac{r}{r-\tau }\right) ^{\alpha } b_{kj}^{m} F_j\right) \right] V(t), $$

where ${\overline{\theta }}\in [-\tau ,t]$ such that ${\overline{V}}(t)=(t-t_0+{\overline{\theta }}+r)^{\alpha } {\overline{W}}(t)$, when ${\overline{V}}(t)=V(t)$.

From (34) and (36), we have

37 $$\begin{aligned} {}_{t_0}^{C}D_{t}^{\alpha }V(t)&\le -\left[ 1-\sum \limits _{j=1}^{n} \frac{\beta _j}{\beta _k}\left( G_j\left( a_{kj}^{m} +\omega _{kj}\right) +\left( \frac{r}{r-\tau }\right) ^{\alpha }b_{kj}^{m}F_j\right) \right] V(t) +\frac{1+\alpha }{r^{\alpha }\Gamma (2-\alpha )}{\overline{V}}(t)\nonumber \\&\le \left\{ -\left[ 1-\sum \limits _{j=1}^{n}\frac{\beta _j}{\beta _k} \left( G_j\left( a_{kj}^{m}+\omega _{kj}\right) +\left( \frac{r}{r-\tau }\right) ^{\alpha }b_{kj}^{m}F_j\right) \right] +\frac{1+\alpha }{r^{\alpha }\Gamma (2-\alpha )}\right\} V(t), \end{aligned}$$

when $${\overline{V}}(t)=V(t)$$.

From (31), it follows that

38 $$ {}_{t_0}^{C}D_{t}^{\alpha }{\overline{V}}(t)\le 0, $$

for $$t\ge t_0$$.

On the basis of Definition 3, we get

39 $$ {\overline{V}}(t)\le {\overline{V}}(t_0), $$

for $t\ge t_0$. Hence for $i=1,2,\ldots ,n,$

40 $$ |x_i(t)|\le \beta _iW(t)\le \frac{\beta _{i}r^{\alpha }\Vert \varphi \Vert _C}{\beta _{\min }(t-t_0+r)^{\alpha }}, $$

where $\beta _{\min }=\min \{\beta _i, i=1,2,\ldots ,n\}$, for $t\ge t_0$, which implies

$$ \Vert x(t)\Vert \le \frac{\Theta r^{\alpha }\Vert \varphi \Vert _C}{(t-t_0+r)^{\alpha }}, $$

where $\Theta =\frac{1}{\beta _{\min }}\sum \nolimits _{i=1}^{n}\beta _i$. Therefore, FMDNNs (1) can be achieved global $O(t^{-\alpha })$ stabilization under the designed control law (30). $\square$

In the following, we propose the following output feedback control rule with time delays:

41 $$ u_i(t)=\sum \limits _{j=1}^{n}\omega _{ij}g_j(x_j(t)) +\sum \limits _{j=1}^{n}\rho _{ij}f_j(x_j(t-\tau (t))), $$

for $$i=1,2,\ldots ,n$$.

#### **Theorem 4**

*FMDNNs* (1) *with the output feedback control rule* (41) *can be achieved global*$$O(t^{-\alpha })$$*stabilization for any*$$\varphi \in C_{\tau }$$*if there exist a constant*$$r>\tau $$*andnpositive constants*$$\beta _i$$$$(i=1,2,\ldots ,n)$$*such that*

42 $$ \sum \limits _{j=1}^{n} \beta _j \left( G_{j}\omega _{ij}+\left( \frac{r}{r-\tau }\right) ^{\alpha }F_{j} \rho _{ij}\right)\le {} \beta _i \left( 1-\frac{1+\alpha }{r^{\alpha }\Gamma (2-\alpha )}\right)  -\sum \limits _{j=1}^{n} \beta _j \left( a_{ij}^{m}G_j+\left( \frac{r}{r-\tau }\right) ^{\alpha }b_{ij}^{m}F_j\right) ,$$

*for all*$$i=1,2,\ldots ,n.$$

#### *Proof*

Define two Lyapunov functions as follows:

43 $$\begin{aligned} \left\{ \begin{array}{l} W(t)=\max \left\{ \frac{|x_i(t)|}{\beta _i},i=1,2,\ldots ,n \right\} ,\\ V(t)=(t-t_0+r)^\alpha W(t), \end{array} \right. \end{aligned}$$

and let

44 $$\begin{aligned} \left\{ \begin{array}{l} {\overline{W}}(t)=\sup \nolimits _{-\tau \le \theta \le t} W(\theta ), \\ {\overline{V}}(t)=\sup \nolimits _{-\tau \le \theta \le t} V(\theta ), \end{array} \right. \end{aligned}$$

for $$t\ge t_0$$.

Through Theorem 1, we have

45 $$ {}_{t_0}^{C}D_{t}^{\alpha }V(t) \le (t-t_0+r)^{\alpha }{}_{t_0}^{C}D_{t}^{\alpha }W(t) +\frac{1+\alpha }{r^{\alpha }\Gamma (2-\alpha )}{\overline{V}}(t). $$

It is obvious that there exists a $k\in \{1,2,\ldots ,n\}$ such that

$$ W(t)=\frac{|x_k(t)|}{\beta _k}, $$

for given $$t\ge t_0$$.

From (43) and (44), we have

46 $$\begin{aligned} {}_{t_0}^{C}D_{t}^{\alpha }W(t)&\le {} \frac{1}{\beta _k}sgn(x_k(t)){}_{t_0}^{C}D_{t_0}^{\alpha }x_k(t)\nonumber \\&\le {} \frac{1}{\beta _k}sgn(x_k(t))\left\{ -x_k(t)+\sum \limits _{j=1}^{n} \gamma _{kj}^{a}(x_j(t))g_j(x_j(t))+\sum \limits _{j=1}^{n} \gamma _{kj}^{b}(x_j(t))f_j(x_j(t-\tau (t)))\right. \nonumber \\&\quad + \left. \sum \limits _{j=1}^{n}\omega _{kj}g_j(x_j(t))+\sum \limits _{j=1}^{n}\rho _{kj}f_j(x_j(t-\tau (t)))\right\} \nonumber \\&\le -\frac{|x_k(t)|}{\beta _k}+\frac{1}{\beta _k}\sum \limits _{j=1}^{n} \beta _j a_{kj}^{m}G_j \frac{|x_j(t)|}{\beta _j}+\frac{1}{\beta _k}\sum \limits _{j=1}^{n} \beta _j b_{kj}^{m} F_j \frac{|x_j(t-\tau (t))|}{\beta _j}\nonumber \\&\quad + \frac{1}{\beta _k} \sum \limits _{j=1}^{n} \beta _j \omega _{kj}G_j \frac{|x_j(t)|}{\beta _j}+\frac{1}{\beta _k}\sum \limits _{j=1}^{n}\beta _j \rho _{kj} F_j \frac{|x_j(t-\tau (t))|}{\beta _j}\nonumber \\&\le -[1-\frac{1}{\beta _k}\sum \limits _{j=1}^{n}\beta _j G_j(a_{kj}^{m}+\omega _{kj})]W(t)+\frac{1}{\beta _k}\sum \limits _{j=1}^{n} \beta _{j} F_j (b_{kj}^{m}+\rho _{kj}){\overline{W}}(t). \end{aligned}$$

Hence

47 $$ (t-t_0+r)^{\alpha }{}_{t_0}^{C}D_{t}^{\alpha }W(t) \le -\left[ 1-\sum \limits _{j=1}^{n} \frac{\beta _j}{\beta _k}\left( G_j\left( a_{kj}^{m} +\omega _{kj}\right)  +\left( \frac{r}{r-\tau }\right) ^{\alpha }F_j\left(b_{kj}^{m}+\rho _{kj}\right) \right) \right] V(t),$$

where ${\overline{\theta }}\in [-\tau ,t]$ such that ${\overline{V}}(t)=(t+{\overline{\theta }}-t_0+r)^{\alpha } {\overline{W}}(t)$, when ${\overline{V}}(t)=V(t)$.

From (45) and (47), we have

48 $$\begin{aligned} {}_{t_0}^{C}D_{t}^{\alpha }V(t)&\le -\left[ 1-\sum \limits _{j=1}^{n} \frac{\beta _j}{\beta _k}\left( G_j\left( a_{kj}^{m}+\omega _{kj}\right) +\left( \frac{r}{r-\tau }\right) ^{\alpha }F_j\left( b_{kj}^{m}+\rho _{kj}\right) \right) \right] V(t) +\frac{1+\alpha }{r^{\alpha }\Gamma (2-\alpha )}{\overline{V}}(t)\nonumber \\&\le \left\{ -\left[ 1-\sum \limits _{j=1}^{n} \frac{\beta _j}{\beta _k}\left( G_j\left( a_{kj}^{m}+\omega _{kj}\right) +\left( \frac{r}{r-\tau }\right) ^{\alpha }F_j\left( b_{kj}^{m}+\rho _{kj}\right) \right) \right] +\frac{1+\alpha }{r^{\alpha }\Gamma (2-\alpha )}\right\} V(t), \end{aligned}$$

when $${\overline{V}}(t)=V(t)$$.

It follows that

49 $$ {}_{t_0}^{C}D_{t}^{\alpha } {\overline{V}}(t)\le 0, $$

for all $$t \ge t_0$$.

On the basis of Definition 3, we get

50 $$ {\overline{V}}(t)\le {\overline{V}}(t_0), $$

for $$t\ge t_0$$. Hence $$i=1,2,\ldots ,n$$,

51 $$ |x_i(t)|\le \beta _i W(t)\le \frac{\beta _i r^{\alpha }\Vert \varphi \Vert _C}{\beta _{\min }(t-t_0+r)^{\alpha }}, $$

where $\beta _{\min }=\min \{\beta _i, i=1,2,\ldots ,n\}$, for $t\ge t_0$, it follows

$$ \Vert x(t)\Vert \le \frac{\Theta r^{\alpha }\Vert \varphi \Vert _C}{(t-t_0+r)^{\alpha }}, $$

where $\Theta =\frac{1}{\beta _{\min }}\sum \nolimits _{i=1}^{n}\beta _i$. Therefore, FMDNNs (1) can be achieved global $O(t^{-\alpha })$ stabilization under the designed control law (41). $\square$

#### *Remark 3*

It needs to point out that fractional-order systems can be said rarely exponential stability. While, global Mittag–Leffler stability or global $O(t^{-\alpha })$ stability can be used to describe asymptotic stability of fractional-order systems. In consideration of the complex and rich nonlinear behaviors of fractional-order systems, especially, for the fractional-order systems with time delays, we employ global $O(t^{-\alpha })$ stabilization for a class of FMDNNs in Theorems 1–4.

#### *Remark 4*

As a useful tool, Lyapunov function method has been introduced to fractional-order systems by borrowing ideas from classical Lyapunov function method in integer-order systems. In Theorems 1–4, a class of new fractional Lyapunov functions have been established, which consist of two Lyapunov functions [i.e., time-invariant Lyapunov function *W*(*t*) and time-varying Lyapunov function *V*(*t*)]. For this structure of Lyapunov functions, we can regard the Caputo derivative of *V*(*t*) as two parts which can be estimated by means of Leibniz rule.

## Numerical examples

In this section, two numerical examples are given to show the effectiveness of the proposed theoretical results.

### *Example 1*

Consider a two-dimensional FMDNNs as follows:

52 $$\begin{aligned} \left\{ \begin{array}{l} {}_{t_0}^{C}D_{t}^{\alpha }x_1(t)=-x_1(t) +a_{11}(x_1(t))g_1(x_1(t))+a_{12}(x_2(t))g_2(x_2(t))\\ \quad \quad \quad \quad \quad  +b_{11}(x_1(t))f_1(x_1(t-\tau ))+b_{12}(x_2(t))f_2(x_2(t-\tau ))+u_1(t),\\ {}_{t_0}^{C}D_{t}^{\alpha }x_2(t)=-x_2(t) +a_{21}(x_1(t))g_1(x_1(t))+a_{22}(x_2(t))g_2(x_2(t))\\ \quad \quad \quad \quad \quad  +b_{21}(x_1(t))f_1(x_1(t-\tau ))+b_{22}(x_2(t))f_2(x_2(t-\tau ))+u_2(t),\\ \end{array} \right. \end{aligned}$$

where $$\alpha =0.95$$, $$\tau =1$$, $$t_0=0$$, $$g_j(\chi )=f_j(\chi )=\tanh (\chi )$$$$(j=1,2)$$, and

$$\begin{aligned}&a_{11}(x_1)=\left\{ \begin{array}{ll} 1.5,&{}\quad |x_1|>1,\\ 2.0, &{}\quad |x_1|< 1,\end{array}\right. \quad a_{12}(x_2)=\left\{ \begin{array}{ll} 0.1,&{}\quad |x_2|>1,\\ 0.2, &{}\quad |x_2|< 1,\end{array}\right. \quad a_{21}(x_1)=\left\{ \begin{array}{ll} 0.5,&{}\quad |x_1|>1,\\ 0.4, &{}\quad |x_1|< 1,\end{array}\right. \\&a_{22}(x_2)=\left\{ \begin{array}{ll} 1.8,&{}\quad |x_2|>1,\\ 1.5, &{}\quad |x_2|< 1,\end{array}\right. \quad b_{11}(x_1)=\left\{ \begin{array}{ll} -3.5,&{}\quad |x_1|>1,\\ -4.0, &{}\quad |x_1|< 1,\end{array}\right. \quad b_{12}(x_2)=\left\{ \begin{array}{ll} -1.8,&{}\quad |x_2|>1,\\ -1.5, &{}\quad |x_2|< 1,\end{array}\right. \\&b_{21}(x_1)=\left\{ \begin{array}{ll} 1.2,&{}\quad |x_1|>1,\\ 1.0, &{}\quad |x_1|< 1,\end{array}\right. \quad b_{22}(x_2)=\left\{ \begin{array}{ll} -1.8,&{}\quad |x_2|>1.\\ -1.5, &{}\quad |x_2|< 1.\end{array}\right. \end{aligned}$$

It is obvious that we can get $G_j=F_j=1$, $j=1,2.$

**Fig. 1:**
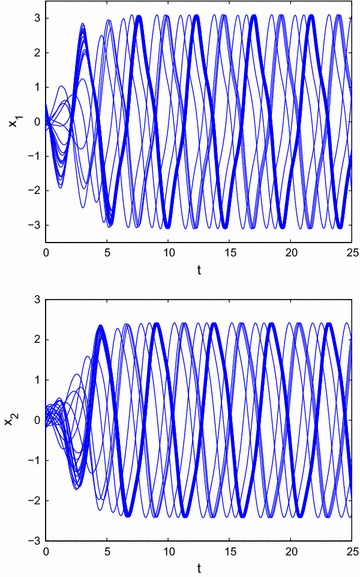
Transient behavior of $x_{1}(t)$ and $x_{2}(t)$ for (52) without external controller

Figure 1 shows the results of time response of (52) without external controller, which implies that the state trajectory of (52) can not convergence to the origin.

Assume that there exist two positive constants $\beta _1$ and $\beta _2$ to satisfy

53 $$\begin{aligned} \left\{ \begin{array}{l} \beta _1 p_{11}+\beta _2 p_{12} \le \beta _1\left( 1-\left( a_{11}^{m}G_1+b_{11}^{m}F_1\right) \right) +\beta _2 \left( a_{12}^{m}G_2+b_{12}^{m}F_2\right) ,\\ \beta _2 p_{22}+\beta _1 p_{21} \le \beta _2 \left( 1-\left( a_{22}^{m}G_2+b_{22}^{m}F_2\right) \right) +\beta _1 \left( a_{21}^{m}G_1+b_{21}^{m}F_1\right) , \end{array}\right. \end{aligned}$$

it follows that there exists a positive constant *r* such that

$$\begin{aligned} \left\{ \begin{array}{l} \beta _1 p_{11}+\beta _2 p_{12} \le \beta _1\left( 1-\frac{1+\alpha }{r^{\alpha }\Gamma (2-\alpha )}-\left( a_{11}^{m}G_1 +\left( \frac{r}{r-\tau }\right) ^{\alpha }b_{11}^{m}F_1\right) \right) -\beta _2 \left( a_{12}^{m}G_2+\left( \frac{r}{r-\tau }\right) ^{\alpha } b_{12}^{m}F_2\right) ,\\ \beta _2 p_{22}+\beta _1 p_{21} \le \beta _2 \left( 1-\frac{1+\alpha }{r^{\alpha }\Gamma (2-\alpha )}- \left( a_{22}^{m}G_2+\left( \frac{r}{r-\tau }\right) ^{\alpha }b_{22}^{m}F_2\right) \right) -\beta _1 \left( a_{21}^{m}G_1+\left( \frac{r}{r-\tau }\right) ^{\alpha } b_{21}^{m}F_1\right) , \end{array}\right. \end{aligned}$$

which implies the conditions of Theorem 1 hold.

From Example 1, we have

$$\begin{aligned} \left\{ \begin{array}{l} \beta _1 (p_{11}+5)+\beta _2 (p_{12}+2) \le 0.\\ \beta _2 (p_{22}+2.6)+\beta _1 (p_{21}+1.7) \le 0, \end{array}\right. \end{aligned}$$

then we can choose $p_{11}=-5$, $p_{12}=-2$, $p_{21}=-2$, $p_{22}=-3$, i.e., the state feedback controller without time delays can be designed as follows:

54 $$\begin{aligned} \left\{ \begin{array}{l} u_1(t)=-5x_1(t)-2x_2(t).\\ u_2(t)=-2x_1(t)-3x_2(t). \end{array}\right. \end{aligned}$$

**Fig. 2:**
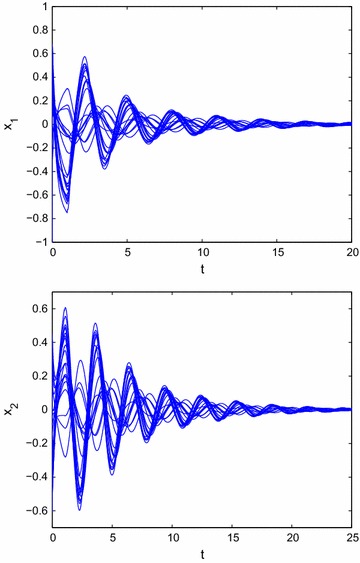
Transient behavior of $x_{1}(t)$ and $x_{2}(t)$ for (52) with state feedback control rule $u_1(t)=-5x_1(t)-2x_2(t), u_2(t)=-2x_1(t)-3x_2(t)$

According to Theorem 1, system (52) can be achieved global $O(t^{-\alpha })$ stabilization. From Fig. 2, we can get that the state trajectory of the resulting closed-loop system of (52) with the designed control law (54) is globally $O(t^{-\alpha })$ stable.

Similarly, select the state feedback controller with time delays designed as follows:

55 $$\begin{aligned} \left\{ \begin{array}{l} u_1(t)=-7x_1(t)-3x_2(t)+0.5x_1(t-1)-0.1x_2(t-1).\\ u_2(t)=-3x_1(t)-5x_2(t)+0.1x_1(t-1)-0.5x_2(t-1). \end{array}\right. \end{aligned}$$

**Fig. 3:**
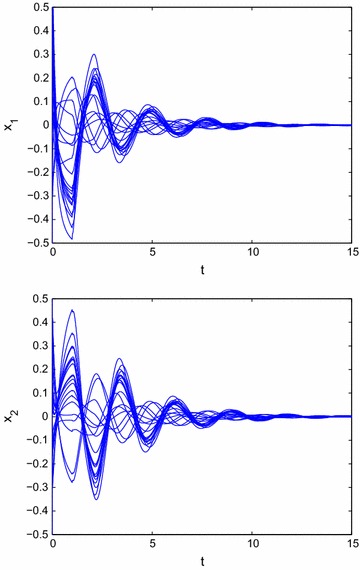
Transient behavior of $x_{1}(t)$ and $x_{2}(t)$ for (52) with state feedback control rule $u_1(t)=-7x_1(t)-3x_2(t)+0.5x_1(t-1)-0.1x_2(t-1)$, $u_2(t)=-3x_1(t)-5x_2(t)+0.1x_1(t-1)-0.5x_2(t-1)$

Then it follows from Theorem 2 that system (52) can be achieved global $O(t^{-\alpha })$ stabilization. From Fig. 3, we can get that the state trajectory of the resulting closed-loop system of (52) with the designed control law (55) is globally $O(t^{-\alpha })$ stable.

### *Example 2*

Consider an one-dimensional FMDNNs as follows:

56 $$ {}_{t_0}^{C}D_{t}^{\alpha }x(t)=-x(t)+a(x(t))g(x(t)) +b(x(t))f(x(t-\tau ))+u(t), $$

where $$\alpha =0.5$$, $$\tau =1$$, $$t_0=0$$, $$g(\chi )=\sin (\chi )$$, $$f(\chi )=\tanh (\chi )$$, and

$$\begin{aligned} a(x)=\left\{ \begin{array}{ll} 1.0,&{}\quad |x|>1,\\ 1.2, &{}\quad |x|< 1,\end{array}\right. \quad b(x)=\left\{ \begin{array}{ll} -4.8,&{}\quad |x|>1.\\ -3.5, &{}\quad |x|< 1.\end{array}\right. \end{aligned}$$

It is obvious that we can obtain $G=F=1.$

**Fig. 4:**
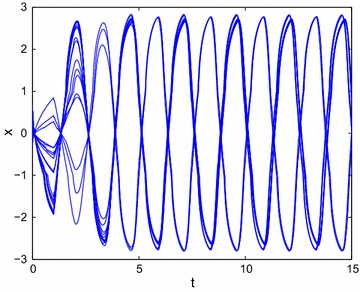
Transient behavior of *x*(*t*) for (56) without external controller

Figure 4 shows the results of time response of (56) without external controller, which implies that the state trajectory of (56) can not convergence to the origin.

In order to apply Theorem 3, the following inequality needs to be satisfied

57 $$ \omega +5 \le 0, $$

then we can choose $\omega$ = −5, i.e., the state feedback controller without time delays can be designed as follows:

58 $$ u(t)=-5\sin (x). $$

**Fig. 5:**
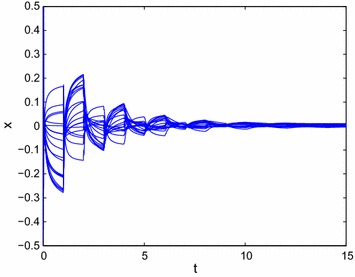
Transient behavior of *x*(*t*) for (56) with output feedback control rule $u(t)=-5\sin (x)$

It follows from Theorem 3 that system (56) can be achieved global $O(t^{-\alpha })$ stabilization. From Fig. 5, we can get that the state trajectory of the resulting closed-loop system of (56) with the designed control law (58) is globally $O(t^{-\alpha })$ stable.

Similarly, select the output feedback controller with time delays designed as follows:

59 $$ u(t)=-7\sin (x)-\tanh (x-1). $$

**Fig. 6:**
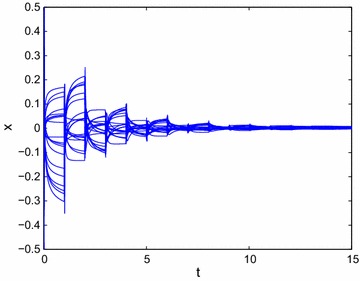
Transient behavior of *x*(*t*) for (56) with output feedback control rule $u(t)=-7\sin (x)-\tanh (x-1)$

Then it follows from Theorem 4 that system (56) can be achieved global $O(t^{-\alpha })$ stabilization. From Fig. 6, we can get that the state trajectory of the resulting closed-loop system of (56) with the designed control law (59) is globally $O(t^{-\alpha })$ stable.

## Concluding remarks

In this article, we exploit the global $O(t^{-\alpha })$ stabilization for a class of fractional-order memristive neural networks with time delays. The main theoretical results of this article are that the linear state feedback control law and the output feedback control law are constructed to stabilize the fractional systems. In addition, some sufficient conditions ensuring to stabilize fractional-order systems are also given in terms of algebraic inequalities according to a new fractional Lyapunov function and a fractional-order differential inequality skill. The article provides a novel way to construct a Lyapunov function and a new method to deal with fractional-order inequalities, which may be applied to discuss other properties or analyze other more complex systems such as the fractional-order form of the model explored in the literatures Chandrasekar and Rakkiyappan (2016), Lou et al. (2013), Shang (2014, 2015, 2016), Wang et al. (2003), Yang and Tong (2016) and so on. Future research will focus on these issues.
